# Marine Peptides: Bioactivities and Applications

**DOI:** 10.3390/md13074006

**Published:** 2015-06-29

**Authors:** Randy Chi Fai Cheung, Tzi Bun Ng, Jack Ho Wong

**Affiliations:** School of Biomedical Sciences, Faculty of Medicine, the Chinese University of Hong Kong, Hong Kong, China

**Keywords:** bioactive, marine peptides, nutraceuticals, pharmaceuticals

## Abstract

Peptides are important bioactive natural products which are present in many marine species. These marine peptides have high potential nutraceutical and medicinal values because of their broad spectra of bioactivities. Their antimicrobial, antiviral, antitumor, antioxidative, cardioprotective (antihypertensive, antiatherosclerotic and anticoagulant), immunomodulatory, analgesic, anxiolytic anti-diabetic, appetite suppressing and neuroprotective activities have attracted the attention of the pharmaceutical industry, which attempts to design them for use in the treatment or prevention of various diseases. Some marine peptides or their derivatives have high commercial values and had reached the pharmaceutical and nutraceutical markets. A large number of them are already in different phases of the clinical and preclinical pipeline. This review highlights the recent research in marine peptides and the trends and prospects for the future, with special emphasis on nutraceutical and pharmaceutical development into marketed products.

## 1. Introduction

Around 70% of the Earth’s surface is covered by the oceans which occupy 90% of the biosphere. Marine species make up around half of the total global biodiversity and they have been extensively explored in the last decades for potential sources of novel bioactive natural products. Because of the difficulties in exploring deep water habitats, many bioactive natural products have yet to be isolated, identified and characterized, thus the oceans constitute a rich source of novel compounds. Peptides are important bioactive natural products which are present in many marine species and extensive research has been conducted on them [1]. In most cases, the origins and roles of bioactive peptides in marine species are uncertain. Their strong bioactivities do not relate to their *in situ* roles, such as antitumor, antidiabetic, neroprotective and cardioprotective actions [2]. The discovery of these bioregulatory roles together with elucidation of the mechanisms of action of the marine peptides would advocate the peptides to be used as potential drugs for cancer, diabetes or hypertension treatment.

Bioactive peptides were first discovered and isolated in marine species as neurotoxin [3], cardiotonic peptide [4], antiviral and antitumor peptide [5], cardiotoxin [6] and antimicrobial peptide [7]. Since then, the investigations on marine bioactive peptides have continued with intent to also ascertain their applications. The broad bioactivity spectrum of marine peptides has high potential nutraceutical and medicinal values which attract the attention of the pharmaceutical and nutraceutical industry, hoping that they can be used in treatment or prevention of various diseases. Nutraceutical is a word formed by the combination of “nutrition” and “pharmaceutical”. In recent years, substantial research efforts were dedicated to this area and it was projected that the global nutraceutical market would reach USD 250 billion by 2018 [8]. Nutraceuticals or health promoting products are food-derived components (naturally occurring or enzymatically generated) that, in addition to their nutritional value exert a physiological effect on the body [9]. They are usually claimed to prevent chronic diseases, enhance the immune system, manage stressful conditions, control body weight, regulates the blood glucose level, improve cognitive function, delay the aging process or increase life expectancy, *etc.* Bioactive peptides are commonly incorporated into nutraceutical products. They are transformed into the active form after gastrointestinal digestion, absorbed through the intestine, and transported to the circulatory system to exert their diverse bioactivities [10].

There are two representative marine peptide-derived pharmaceutical products, ziconotide (see Table 1) and brentuximab vedotin, which are a natural marine peptide and a peptide derivative, respectively, have reached the market. Both of them are manufactured by chemical synthesis. Ziconotide (Prialt^®^), a peptide found in marine cone snail, was the first marine peptide approved by FDA in 2004 for analgesic use [11]. Then, in 2011, another marine peptide-derived drug (Adcetris^®^) from sea hare was approved by FDA for cancer treatment. Several other marine peptide-derived compounds are currently being assessed in different phases of clinical trials in the United States and Europe. They included plitidepsin (see Table 1) and glembatumumab vedotin which is use for treatment of various cancers [12]. Dermochlorella^®^ DG in which its active ingredient is oligopeptide purified from algae. It came into cosmetic market as skin firmer and toner [13]. Katsuobushi oligopeptide (see Table 1) is a linear pentapeptide isolated from dried bonito (katsuobushi) was found to exhibit angiotensin-I converting enzyme inhibitory activity. Some antihypertensive capsules which are sold as nutraceuticals contain the peptide as one of the components [14]. Gelatin is a polypeptide obtained from animal collagen by hydrolytic degradation. It usually serves as a protein supplement in nutraceutical and food industries. Gelatin is conventionally extracted from the skin and bone collagens of cows and pigs. Consumers raised concern about its safety especially when there was an outbreak of mad cow disease in the 1980s. Besides, from the perspectives of ethics and religion, fish gelatin was promoted as a better alternative to traditional gelatin products made from cows and pigs. It is a protein-rich source and usually included in muscle-building diets [15]. Another related product containing a mixture of tripeptides, dipeptides and free amino acids obtained from the collagen hydrolysate is marketed as nutraceuticals for the maintenance of healthy bones [16]. It is quite common for people in USA and Europe to take nutraceuticals or dietary supplements with anxiolytic properties. They contain marine-derived hydrolysates which contain opioid-like peptides as the active ingredients [17]. There are several commercial products available in Europe and the USA such as Stabilium^®^, Gabolysat^®^ PC60, Protizen^®^ and Procalm^®^. However, there are only a few clinical studies about the opioid-like effect and anxiolytic properties of these marine-protein hydrolysates. Marine bioactive peptides can be released from protein by fermentation. A commercially available fermented fish protein concentrate Seacure^®^ was found to modulate the mucosal immune response and induce biological repair-promoting response in the murine gut model [18]. The two products from marine protein hydrolysates, Nutripeptin^®^ and Hydro MN Peptide^®^, were capable of lowering the postprandial blood glucose level and alleviating type II diabetes symptoms. Nutripeptin^®^ is manufactured by enzymatic hydrolysis of fish fillet or fish muscle protein [19]. One of the components of Hydro MN Peptide^®^ is Peptide N^®^ which is a marine protein hydrolysate [20]. Both products have also been claimed to prevent body fat deposition and thus aid in weight management [19,20]. Table 1 summarizes the structures or amino acid sequences of selected marine peptides with different bioactivities.

With the advancement of marine peptides to the current preclinical and clinical pipeline, their contribution to the future pharmacopeia seems to be promising. New technologies and close collaborations between institutional and industrial investigators will be crucial to ensure the future success of marine peptides as novel therapeutics that can make a vital contribution to the treatment or prevention of various diseases [21].

**Table 1:** Structures or amino acid sequences of selected marine peptides with different bioactivities.

Peptides	IUPAC Names/Amino Acid Sequences
ziconotide	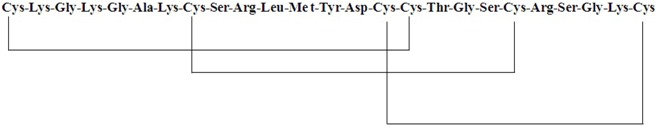
brentuximab vedotin	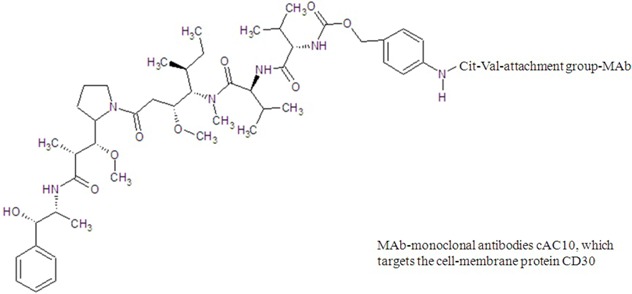
glembatumumab vedotin	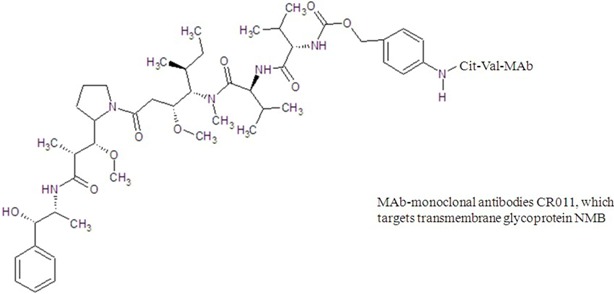
plitidepsin	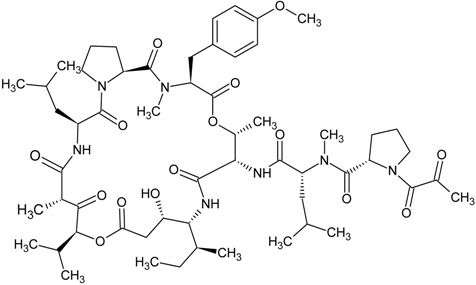
Katsuobushi oligopeptide	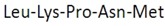
homophymine A	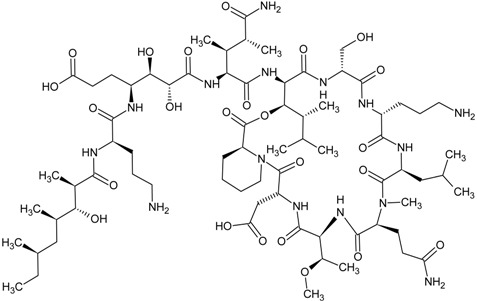
dolastatin 10	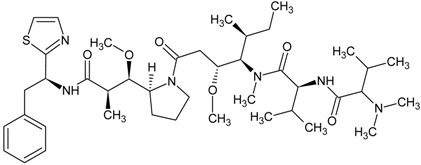
dolastatin 15	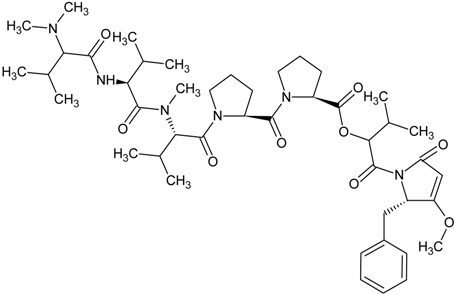
hemiasterlin	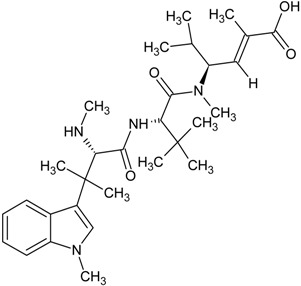
monomethyl auristatin E	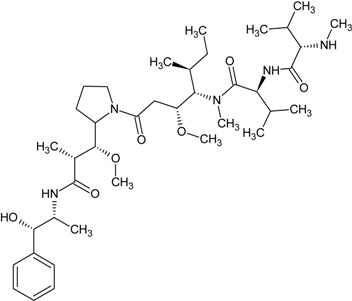
HTI-286	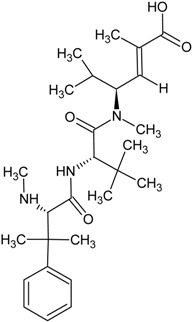
didemnin B	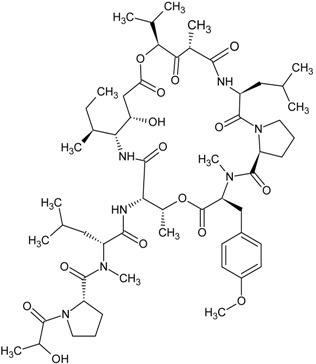
Pardaxin	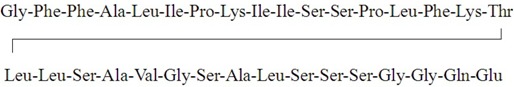

## 2. Procedure for Isolating Marine Peptides

In a typical procedure for discovery of marine bioactive (linear or cyclic) peptides, the peptides are firstly extracted from the sources. The extract is screened for a specific bioactivity, fractionated using a bioassay-guided fractionation procedure, and finally purified to yield a single bioactive peptide. In general, organic solvents like methanol or ethyl acetate are usually used in first step of cyclic peptide extraction. Then the methanol or ethyl acetate extract is concentrated and partitioned with other solvents like hexane, carbon tetrachloride or dichloromethane. The partially purified extract is subjected to silica gel or size exclusion chromatography and the product is eluted with solvents of increasing polarity. Reversed phase C18 HPLC is used in the final purification step [22,23]. For linear peptides, the general procedure usually includes size exclusion chromatography, reverse-phase HPLC and ion exchange chromatography. The aqueous extract is first size-fractionated using a size exclusion chromatography column. The target fraction is concentrated, applied on a cation/anion exchange chromatography column and eluted in accordance with the basicity of the peptide. Reverse phase C18 HPLC with an additional step of rechromatography on the same column for excluding impurities will be used for final purification [24,25].

Bioactive peptides could also be obtained from digestion of proteins of the marine organisms. The most commonly used digestive enzymes are pepsin, trypsin, α-chymotrypsin, papain and some commercial protease cocktails [26]. The hydrolysates are screened for various bioactivities after digestion and fractionated according to size by ultrafiltration [27]. The fraction that shows the highest bioactivity is then further resolved to individual peptides using reverse phase high performance liquid chromatography or size exclusion chromatography. Finally the individual peptide fractions are identified using the combined techniques of mass spectrometry and protein sequencing [28].

The results of some isolations from marine origins showed that the crude extracts or purified fractions contained structurally uncharacterized compounds (probably peptides) exhibited promising *in vitro* or *in vivo* bioactivities and they still deserve further investigations [29].

Bioactive peptides or protein hydrolysates can be extracted and isolated from the protein of the marine species by various methods in industrial-scale production. Organic solvent extraction method was used traditionally, but it is a time-consuming, expensive and environmental unfriendly technique. Nowadays, better extraction techniques like supercritical fluid extraction, pressurized solvent extraction, microwave-assisted extraction, ultrasound-assisted extraction, pulsed electric field-assisted extraction and enzyme-assisted extraction are preferred [30]. After the extraction procedure, the proteins are subjected to hydrolysis by which the proteins are hydrolyzed into bioactive peptides. Enzymatic hydrolysis is preferred in the nutraceutical and pharmaceutical industries in order to avoid harsh chemical and physical treatment [31] and preserve the functionality and nutritive values [32]. The hydrolysis products, bioactive peptides, are further concentrated and separated according to their different molecular weights by membrane filtration. Usually an ultrafiltration membrane with a molecular weight cutoff (MWCO) at 20 kDa or above is used to separate peptides from unhydrolyzed proteins. Membranes with a MWCO at 4–8 kDa are suitable for fractionation of bioactive peptides with desired molecular weights. Membranes with a low MWCO at around 0.2 kDa are used to concentrate the peptide. Diafiltration using a nanofiltration membrane is employed to desalt, deodorize and decolorize the peptide solution [33,34]. A novel ultrafiltration membrane bioreactor technology has recently emerged. It is possible to obtain sequential enzymatic digestions from the marine protein in a system by multistep recycling membrane reactor combined with an ultrafiltration membrane system to fractionate marine hydrolysates according to different molecular weight ranges [35]. Finally, more sophisticated techniques mentioned in the previous paragraphs can be used for further purification and characterization of the bioactive peptide.

## 3. Marine Peptides with Different Bioactivities

### 3.1. Antimicrobial Peptides

Antimicrobial peptides (AMPs) play a crucial part in the innate immunity and can be regarded as host defensive peptides. They are usually amphiphilic, have high cysteine content and are positively charged in their active forms. The AMPs can protect the hosts against a broad range of pathogenic infections which makes them attractive as therapeutic agents [36]. Marine environments are remarkably different from terrestrial habitats which are more hostile and competitive. Marine organisms live in close proximity with pathogenic microbes [37]. Thus effective protective agents were developed under this aggressive environmental pressure. The marine AMPs were found to be structurally different from their counterparts produced by terrestrial species [38]. They usually have novel structures, and are taxa-specific or even species-specific [39,40]. Marine AMPs are structurally diverse, display a wide spectrum of anti-infective activities, a low bio-deposition rate in body tissues, and are highly specific to targets. They provide different and unique sources of potential anti-infective drug candidates [41]. Cyclic peptides another protein class that came out as a potential natural therapeutics as novel antifungal agent. They are usually isolated from diverse species of marine bacteria and sponges [42].

The antimicrobial peptide epinecidin-1 from grouper (*Epinephelus coioides*) demonstrated antibacterial activity against *Pseudomonas aeruginosa*, *Staphylococcus coagulase*, *Streptococcus pyogenes*, and *Vibrio vulnificus*. Plasmid DNA coding for a green fluorescent protein (EGFP)-epinecidin-1 fusion protein under the control of the cytomegalovirus (CMV) promoter was introduced with electroporation into decapsulated Artemia cysts. The resulting EGFP-epinecidin-1 protein suppressed *V. vulnificus* growth. Zebrafish fed on transgenic Artemia expressing CMV-gfp-epi combined with commercial fodder exhibited enhanced resistance to *V. vulnificus* and an increased survival rate. In addition, feeding of transgenic Artemia to zebrafish affected the immunomodulatory response to *V. vulnificus* (204) infection; expression of immune-responsive genes, including hepcidin and defbl2, was altered, as shown by qPCR. These findings suggest that feeding transgenic Artemia expressing CMV-gfp-epi to larval fish has antimicrobial effects, without the drawbacks of introducing drug residues or inducing bacterial drug resistance [43]. The 6621-Da chitotriosidase-like antimicrobial peptide mytichitin-CB with three disulfide bonds from *Mytilus coruscus* hemolymph exhibited antifungal and antibacterial activities. The mRNA expression level of its precursor mytichitin-1 in the gonad was heightened following bacterial infection signifying a role in the host immune response against intruding bacteria [44]. The structurally unique tongue sole (*Cynoglossus semilaevis*) NK-lysin NKLP27 displayed bactericidal activity dependent on the 5 *C*-terminal residues NKLP27. NKLP27 disrupted bacterial cell membrane integrity, entered into the cytoplasm, and fragmented genomic DNA. NKLP27 curtailed bacterial and viral pathogen dissemination and replication in the fish tissues and elevated expression of immune genes [45]. Some of the marine antimicrobial peptides could be used to combat drug-resistant microbes like tilapia piscidin 4 [46] and epinecidin-1 [47] against *Helicobacter pylori*. The molecular mechanism with which some of these peptides such as peptide H-P-6 (Pro-Gln-Pro-Lys-Val-Leu-Asp-Ser) from microbial hydrolysates of *Chlamydomonas* sp. inhibited *H. pylori* has been elucidated [48].

The polar fraction of the organic extract of the Red Sea sponge *Theonella swinhoei*, contained a bicyclic glycopeptide, theonellamide G. Theonellamide G exhibited strong antifungal activity toward wild and amphotericin B-resistant strains of *Candida albicans* with IC_50_ of 4.49 and 2.0 μM, respectively. It exerted cytotoxicity against the human colon adenocarcinoma cell line (HCT-16) with IC_50_ of 6.0 μM. These data furnish further information pertaining to the different chemical structures and bioactivities of this class of compounds [49]. The bacterium *Bacillus amyloliquefaciens* anti-CA isolated from mangrove system destroyed clinic strains of *Candida albicans*. The bacterial strain anti-CA produced elevated levels of bioactive substance, amylase and protease in the cheap medium containing 2.0% soybean meal, 2.0% wheat flour, pH 6.5 within 26 h. The active principle was a cyclic lipopeptide composed of a heptapeptide and a 15-carbon 3-hydroxy fatty acid. The lipopeptide also destroyed many yeast strains including *C. albicans*, *C. tropicalis*, *Metschnikowia bicuspidata*, *Saccharomyces cerevisiae* and *Yarrowia lipolytica*. Following exposure to the lipopeptide, both the entire cells and protoplasts of *C. albicans* were destroyed [50].

### 3.2. Antiviral Peptides

The marine antiviral peptides are usually found in sponges which are conventionally well-known for their unique bioactive metabolites. The bioactive peptides from sponges are usually cyclic or linear peptides containing atypical amino acids. The peptides are often formed from unusual condensation between amino acids, thus their structures are unique and are rarely found in other terrestrial animals and microbes [1]. They combat virus infection in a number of ways include blocking of virus entry, inhibition of cytopathic effect, viral neutralization, fusion and entry.

The cyclic depsipeptides mirabamides A–D from the marine sponge *Siliquariaspongia mirabilis* suppressed HIV-1 fusion. Mirabamides A–C possess 4-chlorohomoproline and mirabamides A, B and D have an unusual glycosylated amino acid, β-methoxytyrosine 4′-*O*-α-l-rhamnopyranoside, along with an unusual *N*-terminal aliphatic hydroxy acid. Mirabamides A, C and D inhibited HIV-1 in neutralization and fusion assays with IC_50_ values at nM or low μM concentrations Mirabamides A–C suppressed proliferation in *C. albicans* and *B. subtilis* [51]. Later study from the same research group discovered that depsipeptides celebesides and theopapuamides were also isolated from *S. mirabilis*. Celebesides are unique cyclic depsipeptides with a polyketide moiety and five amino acid residues, including a phosphoserine and an uncommon 3-carbamoyl threonine in celebesides A and B. Theopapuamides B–D are undecapeptides with an *N*-terminal fatty acid moiety with 3-acetamido-2-aminopropanoic acid and 4-amino-2,3-dihydroxy-5-methylhexanoic acid. Celebeside A but not celebesides C had HIV-1 neutralizing activity with an IC_50_ value of 1.9 ± 0.4 μg/mL. Theopapuamides A–C inhibited HCT-116 human colon carcinoma cells and displayed potent anti-*Candida albicans* activity [52]. The anti-HIV cyclodepsipeptide, homophymine A (see Table 1) from the marine sponge *Homophymia* sp. possesses 11 amino acid residues and an amide-linked 3-hydroxy-2,4,6-trimethyloctanoic acid moiety and four unusual amino acid residues: l-ThrOMe, (2*R*,3*R*,4*R*)-2-amino-3-hydroxy-4,5-dimethylhexanoic acid, (2*S*,3*S*,4*R*)-3,4-diMe-Gln, and (2*R*,3*R*,4*S*)-4-amino-2,3-dihydroxy-1,7-heptandioic acid. Homophymine A demonstrated a cytoprotective action against HIV-1 infection with an IC_50_ of 75 nM [53].

### 3.3. Antitumor/Cytotoxic Peptides

The antitumor/cytotoxic marine peptides vary considerably in size and structural complexity. They can be classified into four types: (1) linear depsipeptides; (2) cyclic depsipeptides; (3) linear peptides, and (4) marine protein hydrolysates. Many of the depsipeptides contain atypical amino acids with post-translational modifications such as carbamoylation or *N*- and *O*-methylation or amino acids not found in common proteins. Examples of linear depsipeptides include dolastatin 10 (see Table 1), dolastatin 15 (see Table 1), hemiasterlin (see Table 1) and their derivatives like monomethyl auristatin E (see Table 1) and HTI-286 (see Table 1). They exert their cytotoxic action by blocking tubulin polymerization, arresting cell cycle and triggering apoptosis [54,55]. The structural diversity of cyclic depsipeptides is more complex than their linear counterparts. Didemnin B (see Table 1), kahalalide F and their derivatives belong to this class of peptides. Their mechanisms of action are different, and will be discussed in detail in a later section. Pardaxin (see Table 1) is a 33-amino acid peptide which manifests both antimicrobial and antitumor activities. It induced apoptosis by endoplasmic reticulum targeting and c-FOS activation [56]. There is a trend to focus on protein hydrolysates, formed by the enzymatic digestion of marine proteins that are used as antitumor agents. The digested hydrolysates or peptides from oyster extract [57] exhibited *in vivo* antitumor activity in BALB/c mice and tunicate extract [58], tuna dark muscle [59] and squid gelatin [60] demonstrated *in vitro* cytotoxic effect on several human cancer cell lines.

Pardaxin with a lytic action has potent activity against canine perianal gland adenomas and thus has potential veterinary application [61]. *Bullacta exarata* peptides BEPT II and BEPT II-1 manifested potent apoptotic activity toward PC-3 cells [62]. P2, a polypeptide fraction from the traditional Chinese medicine *Arca subcrenata* also exerted an antiproliferative action on HeLa and HT-29 cells without affecting normal hepatocytes. Antitumor effect in S-180 tumor-bearing mice was observed [63]. The cyclic octapeptide reniochalistatin E from the marine sponge *Reniochalina stalagmites* demonstrated cytotoxic activity toward HL-60, HepG2, HeLa, MGC-803, and RPMI-8226 cancer cell lines [64].

### 3.4. Antihypertensive Peptides/Angiotensin-I Converting Enzyme (ACE) Inhibitory Peptides

Hypertension, or high blood pressure, is a chronic medical condition in which the blood pressure in the arteries is elevated. The renin-angiotensin system is one of the endocrine systems for regulating blood pressure. When the blood flow to the kidneys is reduced, renin is secreted and converts angiotensinogen to angiotensin I which is subsequently converted by ACE into the potent vasoconstrictor angiotensin II, resulting in elevated blood pressure. ACE inhibitors block angiotensin II conversion and cause relaxation of blood vessels ensuing in a lower blood pressure [65]. Thus ACE inhibitors can be used as a type of drugs for the treatment of hypertension, but their various side effects [66] has contributed to an increased interest in searching natural or food derived inhibitors. Since the first discovery of naturally peptide [67] and marine peptide [68] with ACE-inhibitory activity, many investigations have been conducted on antihypertensive peptides. The antihypertensive peptides inhibit ACE both competitively and noncompetitively. The mechanism of noncompetitive inhibition has not yet been unraveled and site of the peptides associated with the inhibitory effect often has not been delineated [69]. Structure-activity relationship shows that the binding to ACE is affected by the *C*-terminal tripeptide sequence of the substrate. ACE shows preference to substrates or competitive inhibitors containing hydrophobic amino acid residues at those three positions [70]. The diversity and accessibility of marine sources, especially fish, has led to an intense search and characterization for development as antihypertensive agents in functional foods and nutraceuticals. In fact, a number of commercial products like Calpis, Evolus, BioZate and C12 which contain antihypertensive peptides derived from milk protein are now available in the market [71]. The efficacy of orally administered peptides or proteins depends on their bioavailability for they will be hydrolyzed in the digestive or circulatory system. Thus, potential antihypertensive agents must be resistant to proteases or converted to an active form after digestion and readily absorbed across the intestinal epithelium [72].

Two peptides, MVGSAPGVL (829-Da) and LGPLGHQ (720-Da), with potent antihypertensive and antioxidant activities were produced by hydrolysis of skate (*Okamejei kenojei*) gelatin using Alcalase and protease. The IC_50_ values of their antihypertensive activities were 3.09 and 4.22 μM, respectively. They upregulated the protein and gene expression levels of antioxidant enzymes and manifested free radical-scavenging activity in human endothelial cells [73]. The induction of vasorelaxation in the rat aortas brought about by the angiotensin I converting enzyme inhibiting peptide (Ala-His-Ile-Ile-Ile, 565-Da) from *Styela clava* flesh was prevented by prior treatment with the nitric oxide synthase inhibitor, N(G)-nitro-l-arginine methyl ester (l-NAME). Nitric oxide synthesis and eNOS phosphorylation in human endothelial cells were enhanced and systolic blood pressure in spontaneously hypertensive rats was depressed by the peptide [74]. The major peptides in four fractions (A–D) produced by alcalase hydrolysis of a protein concentrate recovered from a cuttlefish industrial manufacturing effluent with potent ACE inhibitory activity were isolated and identified. However, it remains to be seen which peptides contribute to the potent antihypertensive activity of these fractions [75].

### 3.5. Antioxidant Peptides

Antioxidants are well-known for their beneficial effects on health. They protect the body against reactive oxygen species (ROS), which exert oxidative damage to membrane lipids, protein and DNA. This in turn is associated with cardiovascular disease, diabetes, cancer and Alzheimer’s disease as well as aging. Oxidative stress may play an important part in cardiovascular diseases as it is related to structural and functional changes like endothelial dysfunction, vasoconstriction, atherosclerotic plaque and oxidized low density lipoprotein development which reflect the progression of such diseases [76]. Yet, results of the use of antioxidant therapy for cardiovascular diseases in clinical trials were inconclusive [77]. ROS is believed to promote the onset of type II diabetes by decreasing insulin sensitivity and damaging insulin-producing β-cells in the pancreas [78]. Besides, they also modify the cellular signaling pathways that lead to insulin resistance [79]. Antioxidants have been used in treatments for diabetes-mellitus patients as they can inhibit ROS generation in many different ways, but the results were unsatisfactory. ROS can alter DNA sequences through mutations, deletions, gene amplification, and rearrangements. These changes may trigger some signals that lead to apoptosis or activate some proto-oncogenes and/or inactivate some tumor suppressor genes [80]. That dietary inclusion of antioxidants may contribute to protection against cancer development was found in some epidemiological studies [81]. However, there is no evidence in favor of using antioxidants for cancer prevention in clinical studies [82]. The brain utilizes large amounts of oxygen and is particularly susceptible to oxidative damage. As we become old, the plasma and cellular concentrations of antioxidants gradually declines. The amyloid β-peptides begin to accumulate in the brains of Alzheimer’s disease patients and cause protein oxidation, lipid peroxidation, free radical formation and DNA oxidation. The induced oxidative stress will lead to neurodegeneration and neuronal cell death [83]. It was found that nutritionally-derived antioxidants can protect against amyloid-peptide-induced oxidative stress and neurotoxicity, and delay onset and progression of Alzheimer’s disease [84]. ROS is considered to be a potentially deleterious by-product of aerobic metabolism which may cause oxidative and structural damage to macrobiomolecules. Oxidative stress exists in cells and the accumulation of such damages in a chronic state was proposed to be responsible for the physiological deterioration in aging and finally the death of the organism [85]. Recently, some researchers proposed that aging-associated deterioration is mainly due to gradual pro-oxidizing shift in the redox state of the cells. It causes overoxidation of the redox-sensitive thiol proteins and the subsequent interruption of the redox-regulated signaling pathways [86].

As synthetic antioxidants exhibit some potential health risks, searching natural antioxidants in natural foodstuffs becomes attractive. All organisms have antioxidants to counteract oxidation and its deleterious effect. Dietary intake of antioxidants is now becoming popular and considered as an effective way to fuel up our body with antioxidant reserve [87]. As the synthetic antioxidants exhibit some potential health risks, it is demonstrated once again that the attractiveness of searching natural antioxidants in natural foodstuffs. The exact mechanism of antioxidative activity exhibited by the marine peptides is not fully known. However, several mechanisms have been proposed to elucidate their properties, including metal ion-chelating ability, radical-scavenging activity, and aldehyde adduction [88]. The antioxidative peptides are histidine and hydrophobic residues rich [89]. The high hydrophobicity favors their distribution at the water-lipid interface and enhances the radical-scavenging activity at the lipid phase [90]. The histidine-containing peptides may account for the hydrogen-donating ability, lipid peroxyl radical-trapping ability, and metal-chelating ability from the imidazole group [91].

A trio of antioxidant peptides PC-1 (Tyr-Leu-Met-Ser-Arg, 651.77-Da), PC-2 (Val-Leu-Tyr-Glu-Glu, 668.82-Da), and PC-3 (Met-Ile-Leu-Met-Arg, 662.92-Da) were obtained from croceine croaker (*Pseudosciaena crocea*) muscle using pepsin and alcalase hydrolysis. PC-1 suppressed lipid peroxidation and possessed the best 1,1-diphenyl-2-picrylhydrazyl (DPPH) (EC_50_ value = 1.35 mg/mL), superoxide (EC_50_ value = 0.450 mg/mL), and ABTS (EC_50_ value = 0.312 mg/mL) radical scavenging activities. PC-2 displayed the highest hydroxyl radical scavenging activity (EC_50_ value = 0.353 mg/mL) [92]. The trichloroacetic acid-soluble antioxidative peptides derived from an oyster (*Crassostrea talienwhanensis*) subtilisin digest displayed the amino acid sequences proline-valine-methionine-glycine-aspartic acid (PVMGA, 518-Da) and glutamine-histidine-glycine-valine (QHGV, 440-Da). They had potent DPPH and hydroxyl radical scavenging activities [93]. The 582-Da peptide Gly-Gly-Phe-Asp-Met-Gly derived from the peptic digest of Japanese flounder (*Palatichtys olivaceus*) skin gelatin effectively scavenged intracellular reactive oxygen species and enhanced the expression of catalase, glutathione, and superoxide dismutase thus protecting membrane lipids, DNA and proteins from injury [94].

### 3.6. Cardiovascular Protective Peptides

The cardiovascular protective peptides include bioactive peptides which exhibit activities important to cardiovascular health, including effects on blood pressure, oxidative stress, coagulation, atherosclerosis and lipid metabolism. The first two effects have been discussed previously and this section will illustrate the other bioactivities. The anticoagulant peptide binds to coagulation factors in the blood clotting intrinsic pathway and inhibits their molecular interaction. Besides, they can also antagonize the platelet-membrane glycoprotein integrin and stop platelet aggregation [95]. The anti-atherosclerotic peptides showed their cardiovascular protection effect by suppressing the inflammatory responses in histamine-stimulated endothelial cells that may be related to early atherosclerosis. They down-regulated Egr-1 expression through histamine receptor and PKCδ-dependent MAPKs activation pathway. Thus, the production and expression of cytokine, chemo-attractant, and adhesion molecules were depressed in histamine-stimulated endothelial cells [96]. The hypolipidaemic peptides were shown to have potential functions in modulating endogenous lipid profiles in hyperlipidemic patients. The mechanisms involved in the lipid reduction include binding of bile acid and disruption of cholesterol micelles and their adsorption in the digestive system, and modification of hepatic and adipocytic enzyme activity and gene expression of lipogenic proteins [97]. Up to now, only a few hypolipidaemic peptide sequences have been found. There is a need to characterize the structure of the peptides in order to understand their structure-activity relationship. Fish protein lowers serum cholesterol level by retarding absorption and promoting excretion of cholesterol and bile acid in laboratory animals, attributed to lowered micellar solubility of cholesterol and enhanced bile acid binding capacity [98].

Two peptides P1 (LDAVNR; 686-Da) and P2 (MMLDF; 655-Da) isolated from an enzymatic hydrolysate of *Spirulina maxima* were tested for protective action against endothelial cell activation and early atherosclerosis brought about by histamine, which mediates inflammation, in EA.hy926 endothelial cells. Both undermined the formation and expression of interleukin-6 and MCP-1, the generation of the adhesion molecules P-selectin and E-selectin, thereby attenuating adhesion of monocytes onto endothelial cells. The formation of intracellular reactive oxygen species was suppresses. Egr-1 expression through the histamine receptor and PKCδ-dependent MAPKs activation pathway were inhibited [96]. A 2.5-kDa anticoagulant oligopeptide MEAP isolated from the edible parts of blue mussel (*Mytilus edulis*) exhibited amino acid sequence homology to the EF-hand domain of scallop adductor muscle calmodulin. The thrombin time and activated partial thromboplastin time were extended by MEAP due to interaction with the blood coagulation factors FIX, FX, and FII. Proteolytic activation of FX by the intrinsic tenase complex (FIXa/VIIIa/PLs) and FIIa production from FII by a prothrombinase complex (FXa/FVa/PLs) were inhibited by MEAP [99]. Freshwater clam muscles and whole *Gracilaria* powder (WG) were subjected to hot water extraction. The residual meat was lyophilized, and hydrolyzed at 50 °C by Protamex to yield freshwater clam hydrolyzate (PX5). WG was resolved into two fractions, soluble and insoluble dietary fibre. Bile acid-binding of compounded PX5 and WG produced optimal synergism at a ratio of 1:3 (w/w) and relative bile acid-binding of 45.7%, higher than PX5 and WG alone. Following peptic digestion, the % inhibition of cholesterol micelle formation in cholestyramine, PX5, WG and IDF were 97.6%, 18.5%, 30.8% and 49.3%, respectively [100].

### 3.7. Immunomodulatory Peptides

Some fish hydrolysates showed strong immunomodulatory effects in animals. These effects may be due to enhanced macrophage activity and lymphocyte proliferation, natural killer cell activity and cytokine regulation. However, the specific mechanisms are yet to be solved. The hydrolysate from Atlantic cod promoted the oxidative burst of Atlantic salmon leucocytes and stimulated the bactericidal power of phagocytes [101]. Administration of fermented fish-protein from Pacific whiting and Chum-salmon hydrolysate stimulated T-helper cells to release high levels of cytokines in rats [102]. Yang *et al.* [103] reported that chum salmon hydrolysate stimulated lymphocyte proliferation, cytokine secretion and enhancement of the cytotoxic activity of the natural killer cells in mice. The potential use of these hydrolysates is mainly on aquaculture, as they can enhance the immune system in cultured fish. They are usually added to the fish feed as immunostimulant in controlling infectious diseases in fish farms [104].

Thalassospiramides A and D isolated from cultured *Thalassospira* sp. suppressed lipopolysaccharide-induced nitric oxide production by murine macrophage RAW 264.7 cells [105]. An antiinflammatory tripeptide from salmon pectoral fin byproduct protein hydrolysate downregulated the expression of inducible nitric oxide synthase and cyclooxygenase-2, and hence the formation of nitric oxide and prostaglandin E2. At the same time the levels of pro-inflammatory cytokines, such as interleukin-1β, interleukin-6 and tumor necrosis factor-α were reduced [106]. Two anti-allergic peptides LDAVNR (P1) and MMLDF (P2) from enzymatic hydrolysate of *Spirulina maxima* inhibition reduced histamine release and intracellular Ca^2+^ elevation and thereby suppressed mast-cell degranulation. P1 worked by interfering with signaling pathways dependent on calcium and microtubules. P2 worked by inhibiting phospholipase Cγ activation and reactive oxygen species formation. Nuclear factor-κB translocation and formation of interleukin-4 were undermined by P1 and P2 [107].

### 3.8. Neuropeptides

Some investigations show that the protein hydroylsates or peptides derived from marine species may affect our nervous system after oral intake. They are opioid-like, and exhibit a positive effect on motivation, emotion, behavior, stress, appetite and pain management [17,108,109]. The nutraceutical industry shows interest in these opioid-like peptides and promotes them as dietary supplement for anxiety and stress management. They show similar but weaker effect as endogenous opioid peptides and interact with specific opioid receptors in the nervous, endocrine, immune and digestive systems. They can be used as a natural and safe alternative to opioid drugs which are usually associated with tolerance, dependence and addiction.

The gonadotropin-releasing hormone, which increases the secretion of pituitary gonadotropin has been detected in hagfish [110] and Japanese anchovy [111] brains and in gonads of the prosobranch *Patella caerulea* [112]. Proopiomelanocortin-derived hormones (*i.e.*, corticotropin, melanotropin, *etc.*) from snakeskin gourami (*Trichopodus pectoralis*) [113] and a neuropeptide in marine annelid *Platynereis dumerilii* [114] have been reported.

### 3.9. Neuroprotective Peptide

Marine proteins and peptides suppress the development of neurodegenerative diseases like Parkinson’s disease, Alzheimer’s disease, and multiple sclerosis. Their neuroprotective action is brought about by the direct interaction of absorbed protein and peptide with a variety of cellular and molecular targets with enzyme/ion channels [115].

The neuroprotective peptide (HTP-1) Gly-Thr-Glu-Asp-Glu-Leu-Asp-Lys from the seahorse *Hippocampus trimaculatus* exerted a protective action on PC12 cells and prevented them from the deleterious action of amyloid β42 which is associated with the pathogenesis of Alzheimer’s disease, as evidenced by the enhancement of cell viability and expression of the pro-survival gene (Bcl-2) [116].

### 3.10. Anti-Diabetic Peptide

The development of new therapeutic agents to improve glucose metabolism and prevent and inhibit type-2 diabetes mellitus-related complications is greatly significant. A few fish protein hydrolysates have shown *in vivo* glucose uptake-stimulating activity and could be used in hyperglycaemia management in addition to regular therapy. These glucose uptake stimulating hydrolysates can ameliorate glucose tolerance either by stimulating glucose uptake via a different mechanism to that of insulin or by increasing insulin sensitivity in target cells.

Suppressed fasting blood levels of glycated hemoglobin A1c, glucose, insulin, total triglycerides, free fatty acids, total cholesterol, low density lipoprotein-cholesterol, high-sensitivity C-reactive protein and nitric oxide, but elevated levels of high density lipoprotein-cholesterol, insulin sensitivity index bradykinin, prostacyclin, and adiponectin were noted in Chinese patients with type 2 diabetes mellitus following daily treatment with 13 g marine collagen peptides from fish hydrolysate for 1.5 and 3 months [117]. Treatment of rat model of type 2 diabetes mellitus (established by high fat diet and low doses of streptozotocin) with oligopeptides from marine salmon skin lowered fasting blood glucose level by reducing oxidative stress and inflammation, as evidenced by attenuated levels of serum tumor necrosis factor-α, and interferon-gamma and malondialdehyde, but increased activities of superoxide dismutase and glutathione which exerted anti-apoptotic effect on pancreatic beta-cells [118].

### 3.11. Analgesic Peptides

Conopeptides from marine snail venoms have attracted much interest as leads in drug design. Currently, Prialt^®^ is available as a medication for chronic neuropathic pain. The relatively stable conopeptides obtainable by chemical synthesis target a diversity of transporters, receptors and ion channels. Only a meager number of the predicted constellation of conopeptides have been examined so far, and hence these peptides represent a largely untapped resource for drug discovery. Recent efforts are directed at chemically re-engineering conopeptides to enhance their biopharmaceutical characteristics in order to expedite their clinical application [119]. Cone snails have evolved a myriad of small, stable venom peptides (conopeptides) for defense as well as prey capture. Less than 0.1% has been pharmacologically characterized, but those with elucidated activities target membrane proteins of therapeutic importance, including ion channels, transporters and G protein-coupled receptors. Several conopeptides display antinociceptive activity in animal models, with one of them undergoing clinical development (χ-conopeptide analogue Xen2174) and one marketed (ω-conotoxin MVIIA or Prialt^®^) for the management of severe pain. In addition to their therapeutic potential, conopeptides serve as probes for ascertaining the role of a variety of important membrane proteins in normal and disease physiology [120].

Conotoxins from the worm-hunting cone snails *Conus nux*, *Conus brunneus*, *and Conus princeps* are composed of 7–40 amino acid residues (including 0–5 S-S bonds). The majority of conotoxins affect voltage-gated ion channels, ligand-gated ion channels, G protein-coupled receptors, and neurotransmitter transporters with high affinity and specificity [121]. α-Conotoxin TxIB is a ligand and a valuable therapeutic candidate. It specifically blocks α6/α3β2β3 nicotinic acetylcholine receptors which are potential therapeutic targets for the treatment of addiction and Parkinson’s disease. [122] optimized the cleavage and oxidative folding of α-conotoxin. The peptide toxin from venom of the terebrid marine snail *Terebra variegata* is rich in S-S bonds and demonstrates a distinctive display not found in previously reported snail neuropeptides. It is possible that it targets different neuronal agents with different specificities compared with other snail neuropeptides [123]. Palytoxin is a highly toxic algal biotoxin that converts the Na^+^/K^+^-ATPase into a cationic channel inducing a massive intracellular Na^+^ influx necrosis in skin HaCaT keratinocytes [124]. The peptide neurotoxin Av3 from sea anemone *Anemonia viridis* is specific for arthropod voltage-gated sodium channels [125].

### 3.12. Appetite Suppressing Peptide

Obesity has become a serious public health problem in the developed countries, hence much effort has been made in searching for antiobesity therapeutics. Cholecystokinin and gastrin are peptide hormones linked with the satiety signal and were found to be a potential target [126,127,128]. Some marine peptides were found to be gastrin/cholecystokinin-like or stimulate cholecystokinin release, and thus control appetite. Low-molecular-weight peptides from shrimp head protein hydrolysates have been found to be effective for stimulating cholecystokinin release in STC-1 cells [129]. These peptides are thus suggested as a promising functional food against obesity via regulation of cholecystokinin release.

Marine peptides may be employed as anorexigenic agents and may interact with appetite-suppressing gut hormones including cholecystokinin and glucagon-like peptide 1 to produce anti-obesity effects [130]. Marine peptides may be potentially useful for prevention of metabolic syndrome although clinical studies are [131].

### 3.13. Other Bioactivities

Kazal-type proteinase inhibitor from disk abalone (*Haliotis discus discus*) probably played a role in immune defense and wound healing [132]. The peptide isolated from *Streptomyces* sp. LK3 extract manifested activity against the malaria parasite Plasmodium [133]. Two linear peptidic aspartic protease inhibitors, ahpatinin Ac and ahpatinin Pr, isolated from a marine *Streptomyces* sp. exhibited inhibitory activity toward cathepsin B9 and pepsin [134]. Nazumamide A is a thrombin inhibitor from a sponge-associated marine actinobacterium *Salinispora* sp. [135].

The collagen peptides MCPs from the chum salmon (*Oncorhynchus keta*) exerted optimal anti-inebriating activity in rats before alcohol consumption. They produced their action probably by interfering with alcohol absorption and enhancing alcohol metabolism [136].

Mature *Octopus vulgaris* eggs produce a sperm-activating chemoattractant peptide which enhances sperm motility. Its action is dependent on extracellular calcium ions and phophorylation of tyrosine residues in membrane protein [137].

The insulin-like androgenic gland-specific factor reported in male shrimps, crabs, prawns, crayfish, and spiny lobsters is a masculinizing hormone which is responsible for sex differentiation into males and maintaining the male gonad [138].

The insulin-like peptide in the Eastern rock lobster *Sagmariasus verreauxi*, which is an entity different from the androgenic gland hormone, was expressed in neurosecretory cells of the thoracic ganglia, antennal gland, eyestalk, hepatopancreas, androgenic gland, male and female gonads, male muscle, indicating that it has function(s) other than masculinisation in decapod crustaceans. The similar tissue expression of Drosophila insulin-like peptide 7, together with the phylogenetic clustering of the lobster and fruifly insulin-like peptides, signify that the lobster peptide is a fruifly ortholog. The peptides may find application in enhancing crustacean aquaculture [139].

## 4. Marine Peptide Products in Market and Clinical Trials

As described above in this review, many bioactive marine peptides have been proven to have different bioactivities. Their original or modified form could be utilized as the lead structures for potential nutraceutical and pharmaceutical uses. There is a repertoire of peptides isolated from marine species but only a small portion has been approved for evaluation in the clinical phases and even fewer have managed to reach the market. Table 2 highlights the status of marine peptide products in market and clinical trials. Successful examples comprise ziconotide and brentuximab vedotin, other commercialized examples belong to cosmetics and nutraceuticals. Some peptides are currently being evaluated in different phases in clinical trials such as plitidepsin and CDX011.Some marine peptide candidates that have yielded promising *in vitro* results were subjected to clinical trials, but the investigations have been terminated or discontinued due to lack of efficacy, absence of objective responses or adverse effects experienced in patients. In the following section, some selected examples are used to demonstrate the novel marine peptides discovery, their development and progression to clinical trials as original forms or derivatives.

Ziconotide is a 25-amino acid peptide derived from the ω-conotoxin toxin of *Conus magus* (cone snail found in tropical water) [140]. The fish-hunting snails produce a few conotoxin peptides that act synergistically by targeting the neuromuscular system to immobilize the prey [141]. The ω-conotoxin blocks N-type voltage-sensitive calcium channels and inhibits the pain-related release of neurotransmitters. The nerve signal conduction is inhibited thus resulting in pain relief [142]. As the marine sources cannot provide enough amounts for large-scale production, ziconotide was manufactured by peptide synthesis. The approval by FDA of ziconotide, a synthetic form of ω-conotoxin, as an analgesic agent for amelioration of chronic pain, prompted investigations on other conotoxins, like contulakin G (CGX-1160), for potential therapeutic and clinical usage [143,144].

**Table 2:** The status of marine peptide products in market and clinical trials.

Compound	Natural Product/Derivatives	Source	Applications	Status
ziconotide	natural product	ω-conotoxin toxin from *Conus magus*	analgesics	FDA approved
brentuximab vedotin	derivatives	dolastatin 10 from *Dolabella auricularia*	cancer treatment	FDA approved
glembatumumab vedotin	derivatives	dolastatin 10 from *Dolabella auricularia*	cancer treatment	phase I/II clinical study
katsuobushi oligopeptide	natural product	pentapeptide from hydrolysate of dried bonito	antihypertensive	sold as nutraceuticals
Dermochlorella^®^	natural product	oligopeptide extract from *Chlorella vulgaris*	Skin toner and firmer	sold as skin care product
plitidepsin	natural product	cyclic depsipeptide from *Aplidium albicans*	cancer treatment	phase I/II clinical study
HTI-286	derivatives	hemiasterlin from *Hemiasterella minor*	cancer treatment	preclinical study
kahalalide F	natural product	cyclic tridecapeptide from *Elysia rufescens*	cancer treatment	phase I clinical study
elisidepsin	derivatives	kahalalide F from *Elysia rufescens*	cancer treatment	phase I clinical study
fish gelatin	natural product	hydrolysate of fish collagen and gelatin	nutrient supplements and bone health	sold as nutraceuticals
Gabolysat PC60^®^/Stabilium^®^/Protizen^®^/Procalm^®^	natural product	hydrolysate of fish protein	anxiolytic	sold as nutraceuticals
Seacure^®^	natural product	hydrolysate of fish protein	intestinal health	sold as nutraceuticals
Nutripeptin^®^/Hydro MN Peptide^®^	natural product	hydrolysate of fish protein	postprandial blood glucose control	sold as nutraceuticals

Brentuximab vedotin is an antibody-drug conjugate which targets on cell membrane protein CD30. It consists of (1) a chimeric monoclonal anti-CD30 antibody, brentuximab, linked to (2) a cathepsin-cleavable linker; (3) a para-aminobenzylcarbamate spacer and (4) monomethyl auristatin E (MMAE) which is a strong antimitotic agent. MMAE binds in the vicinity of the vinca peptide site, blocks microtubule assembly and tubulin polymerization [55]. It was approved by FDA in 2011 for treatment of Hodgkin and systemic anaplastic large cell lymphoma. The MMAE is a synthetic analog of dolastatin 10 isolated from *Dolabella auricularia* (sea hare found in the Indian Ocean). The extract from *Dolabella auricularia* was found to be highly efficacious against cancer cells as early as 1972. However, it was not until 1987 that the most potent constituent, dolastatin 10, was isolated and characterized. It is a linear pentapeptide with four distinctive amino acids and exhibited potent inhibitory activity against a battery of human cancer cell lines [145]. However, when it entered clinical trials, the results were unsatisfactory due to lack of efficacy. These disappointing results did not justify further endeavors and consequently dolastatin 10 was withdrawn from antitumor clinical trials [146]. Nevertheless, its synthetic derivatives, MMAE, showed clinically significant activity when it is linked with an antibody that targets CD30 protein [147,148]. The antibody portion attaches to CD30 present on the surface of Hodgkin’s lymphoma cells, delivering MMAE selectively to the tumor cells. Clinical study has started for a similar drug CDX011 which is also known as glembatumumab vedotin, for the treatment of advanced, refractory or resistant breast cancer [149,150]. Its structure contains MMAE links with glembatumumab which targets transmembrane glycoprotein-expressing cancer cells. These similar antibody-drug conjugates, SGN-75, ASG-5ME and soblidotin derived from dolastatin 10 and synthadotin and tasidotin (ILX-651) derived from dolastatin 15, have advanced to Phase I/II/III clinical trials for treatment of cancer, but the developments were suspended by the pharmaceutical company due to “strategic” reasons.

Katsuobushi oligopeptide, a linear pentapeptide from dried bonito (katsuobushi, a traditional Japanese food), was derived from katsuobushi by thermolysin hydrolysis. It manifested ACE inhibitory activity when transformed to the active form in the human digestive system [151]. Katsuobushi oligopeptide exerted antihypertensive activity in patients with hypertension and borderline hypertension in a small-scale clinical trial. Official approval of katsuobushi oligopeptide as foods for specified health use was issued in 1999 by Ministry of Health and Welfare in Japan [152]. Subsequent reports indicated that the peptide brought about relaxation of vascular smooth muscle [153]. They are incorporated in blood pressure-lowering capsules which are sold as nutraceutical [14].

Dermochlorella^®^ is a skin care product which contains *Chlorella vulgaris* (green algae) extract with oligopeptides as the active ingredient. It helps to firm the skin and reduce the colour of the stretch marks. Dermochlorella increases the expression of collagen, elastin, laminin and the inhibitors (elafin and tissue inhibitors of metalloproteinase) of the enzymes that degrade the extracellular matrix and restore skin elasticity [13].

Plitidepsin (also known as dehydrodidemnin B or Aplidin^®^) is a cyclic depsipeptide isolated from *Aplidium albicans* (a tunicate found in Mediterranean Sea) in 1991. It is classified as a didemnin member and its chemical structure closely resembles that of didemnin B which has been submitted to clinical trials for various cancer treatments [154,155,156,157]. Yet, the clinical trials were stopped due to severe fatigue and anaphylaxis experienced in patients [156,158]. Plitidepsin showed comparable levels as didemnin B on the *in vitro* antitumor activity to tumor cell lines [158,159]. Plitidepsin was demonstrated to elicit apoptosis in a cell type- and dose-dependent fashion. These actions are associated with the triggering of early oxidative stress, activation of Rac1 GTPase and suppression of protein phosphatases, which altogether contribute to the sustained activation of JNK and p38 mitogen-activated protein kinases [160]. The natural resource of plitidepsin is limited because of the difficulties in collecting the sacred *Aplidium albicans* and the lack of possible aquaculture or mariculture conditions. The constituent amino acids of plitidepsin are unnatural and heterologus expression is not feasible, and thus they are manufactured by multi-step total synthesis. Phase I/II clinical trials of plitidepsin yielded promising results of antitumor activity in patients with advanced melanoma [161], multiple myeloma [162], non-Hodgkin’s lymphoma [163], advanced medullary thyroid carcinoma [164] and urothelium carcinoma [165].

Hemiasterlin is a linear tripeptide isolated from the sponge *Hemiasterella minor* in 1994. It contains two unique amino acids and exhibited strong cytotoxic activity on the P388 leukaemia cell line [166]. It induces mitotic arrest and apoptosis by blocking mitotic spindle formation and causes tubulin depolymerization [54]. HTI-286 (also known as SPA-110 or taltobulin), a synthetic analogue of hemiasterlin, shows more potent cytotoxic activity than hemiasterlin on human cell lines. The mechanisms of action of both compounds are similar. Preclinical studies showed that HTI-286 inhibited the growth of human tumor xenografts in mice [167]. It has been submitted to phase I clinical trial but unfortunately the results were unsatisfactory. There were no objective responses in patients with advanced solid tumors, besides neutropaenia, nausea, alopecia and pain were observed and further trials were terminated [168]. However, some researchers are still working on this peptide. The combination of antisense oligonucleotides OGX427 and HTI-286 used in therapy intravesically was shown to effectively inhibit orthotopic tumor growth without toxic side effects. The results gave hope to a potential treatment method for bladder cancer [169].

Kahalalide F is a cyclic tridecapeptide that contains several atypical amino-acid residues which showed antitumor activity. It was isolated from *Elysia rufescens* (a sea slug found in Hawaiian water) in 1993 [170]. However, later, it was found that the compound originated from the green alga *Bryopsis* sp., present in the sea slug’s diet [171]. It was active against human cancer cell lines [170,171] and several mechanisms of action have been proposed including an action on lysosomal membrane [172], inhibition of erbB2 tyrosine kinase activity [173], oncosis induction [174], effect on cell membrane permeability [175] and induction of necrosis-like cell death [176], yet no conclusive mechanism has been elucidated. Kahalalide F has entered into clinical trials for the treatment of patients with solid tumors including melanoma, non-small lung cancer and hepatocellular carcinoma [177,178], but the trials came to an end due to lack of antitumor activity. Yet, there is still interest in kahalalide F given that report of a recent investigation in advanced solid tumors therapy in phase I clinical trial has appeared [179]. Elisidepsin (PM02734 or known as Irvalec^®^) is a synthetic kahalalide F derivative which has similar action as kahalalide F. It manifests *in vitro* activity against a variety of tumor cell lines such as breast, colon, pancreas, prostate and lung [180,181,182] as well as *in vivo* activity in xenografted human tumors [183]. These results were promising and gave a rational basis for further investigations such as clinical studies of cancer treatment. A few clinical trials were submitted, they were the study of elisidepsin in patients with advanced solid tumor [184] and the study of combination therapy with erlotinib in patients with advanced malignant solid tumors. The two studies have been completed and the pharmaceutical company decided to suspend the development of elisidepsin in 2012 in view of commercial unviability of this product. However, a phase I study of combination therapy with carboplatin or gemcitabine in patients with advanced malignancies was initiated recently [185]. However the results did not show clinically significant antitumor activity, thus its development program may probably be deferred.

Fish gelatin is derived from hydrolytic degradation of collagen which is generated from seafood processing waste such as skin, bones, scales and fins from fish [15]. It is a valuable protein source and usually incorporated into protein drinks, protein energy bars, muscle-building food and nutritional formula. A similar product, known as collagen peptides, is the protein hydrolysate of collagen or gelatin with a molecular weight around 1–5 kDa. They contain tripeptides, dipeptides and free amino acids that are easily absorbed in the body. Currently, they are marketed as nutraceuticals for the maintenance of normal bone and tendon integrity, improving joint health, brittle nail treatment and scalp hair nourishment [186]. Clinical studies showed that collagen peptides are involved in cartilage matrix synthesis and treatment with the peptides reduced pain in osteoarthritic patients. So they are potential therapeutic agents for osteoarthritis and osteoporosis [16].

Several nutraceuticals or dietary supplements that are marine protein hydrolysates in nature claimed to have anxiolytic properties were commercially available. Gabolysat PC60^®^, a ling fish protein hydrolysate, exhibited diazepam-like effects on stress responsiveness of the rat pituitary-adrenal axis and the sympathoadrenal activity [17]. Stabilium^®^ is another fish protein hydrolysate obtained from enzymatic autolysis of blue ling viscera. It accounted for reducing anxiety in humans and improving memory and concentration in stressed rats [108,109]. Protizen^®^ (a pollock hydrolysate) and Procalm^®^ (a veterinary drug) were reported for similar actions.

Seacure^®^ is a commercial dietary supplement product obtained from proteolysis of Pacific whiting proteins by fermentative yeast. They are packaged as “intestinal health promoting” product. They work directly on animal gastric-damaging models and induce epidermal growth factor-like responses, *i.e.*, stimulate the proliferation of epidermal and epithelial tissues [187]. In a small scale clinical trial, [18] described its prevention and treatment of nonsteroidal anti-inflammatory drugs-induced and other gastrointestinal injurious conditions.

Two commercial products, Nutripeptin^®^ and Hydro MN Peptide^®^, were sold as nutraceuticals for lowering of postprandial blood glucose [19,20]. Nutripeptin^®^ is enzymatic fish protein hydroylsates which can lower and stabilize blood glucose, increases fat burning, and extend the satiety sensation after meals [19]. Its addition to sport drinks has been suggested for improving energy recovery and performance [188]. One of the components of Hydro MN Peptide^®^ is Peptide N^®^ which is a marine protein hydrolysate. Clinical studies found that Peptide N^®^ supplementation facilitated blood glucose stabilization, alleviated obesity risk, mitigated type II diabetes symptoms and induced satiety feeling through its action on metabolic hormone [20].

We have mentioned the large-scale production of protein hydrolysates in the previous section. They are manufactured by protease digestion of the muscles, skin, bones, scales and fins from fish or other marine species in the nutraceutical industry. For the process of manufacture of peptide-based drugs, the drugs are pure compounds rather than a mixture of peptides as in the case of protein hydrolysates. Usually, minute amounts of the marine peptides are present in the marine organisms, and it is difficult to extract enough quantities for commercial use. It is impractical to rely on marine sources to provide the sustainable supply for commercialization [189]. As a result, chemists developed total synthesis approach for production of peptide drugs (solid-phase peptide synthesis of ziconotide [190] and convergent total synthesis of dolastatin 10 [191] and MMAE [192]).

To increase the chance of success in commercializing marine peptides, the best way is to develop close collaborations between academic and industrial partners at the initial stage. Academic institutions can get industrial sponsorship at the early development stage. This approach merges the expertise of academics with knowledge of marine life, pharmacology, analytical and synthetic techniques and the industrial partners with market awareness and business expertise. Starting from the beginning of the marine peptide discovery and development project, like accessibility to marine resources, bioactivity screening, study of the mechanism of action, production of the peptides and recruitment for clinical trials and studies, to the final phase like financial arrangement and marketing strategies, the joint academic-industry venture can push the research project forward, bridge the innovation gap and unlock the potential of the under-exploited ocean habitats. The joint venture benefits both partners that the academia gets knowledge, publications and funding and industry gets new marketable products [13].

## 5. Advances in Selecting Novel Organisms for Marine Peptide Extraction and Future Prospects

Traditional investigations on marine peptides were mainly focused on macroorganisms, like algae, sponges, molluscs, tunicates and fish. However, scale-up extraction or industrial production of the promising peptides derived from these organisms is usually hindered by insufficiency of raw materials [13]. The attention has turned to marine microorganisms for a source of peptides. A sustainable and economic supply of raw materials is often easier to procure for peptides produced by fermentation versus sampling from the environment or culture of macroorganisms displaying a slower growth [193]. Besides, it was discovered that many peptides previously isolated from marine macroorganisms like kahalalide F, dolastatin and its analogs are likely metabolic products of associated microorganisms or microbial origin [171,194,195].

Cryptophycin was first isolated from a marine cyanobacterium, *Nostoc* sp. ATCC 53789 and GSV 224 as a potent fungicide [196]. It belongs to the depsipeptide family and was found later that it bound strongly to the microtubule ends at the vinca-binding domain, and prevented the formation of the mitotic spindle. It exhibited strong cytotoxic activity against drug-resistant human cancer cell lines [197]. Cryptophycin-52, also known as LY355703, is a synthetic analogue of the cryptophycin family produced by total chemical synthesis. In a preclinical study, [198] reported that cryptophycin-52 induced *in vitro* Bcl-2 hyperphosphorylation, cell cycle arrest and growth inhibition in human non-small cell lung carcinoma cells. Phase II clinical trials of cryptophycin-52 yielded promising results of antitumor activity in patients with platinum-resistant advanced ovarian cancer [199] and patients with advanced non-small cell lung cancer [200].

Thiocoraline is a thiodepsipeptide derived from mycelium of the actinomycete *Micromonospora marina* L-13-ACM2-092 [201,202]. It is an antitumor agent with a special mechanism of inhibition of DNA polymerase-α, causing cell cycle arrest and leading to apoptosis [203]. Thiocoraline was found to activate the Notch signaling pathway. It inhibited cell proliferation *in vitro* and induced cell cycle arrest of two neuroendocrine tumor cell lines BON and H727 and slowed down growth in the slow-growing neuroendocrine tumors progression *in vivo* [204]. William and his colleagues isolated two novel linear peptides from the marine cyanobacterium *Symploca* sp. in 2002 and 2003 respectively. They are known as tasiamide (hexapeptide) [205] and tasiamide B (octapeptide) [206]. Tasiamide B contains a unique amino acid residue, 4-amino-3-hydroxy-5-phenylpentanoic acid and both peptides showed cytotoxic activity against human nasopharyngeal carcinoma cell lines. Later reports demonstrated that the synthetic analogues of tasiamide showed inhibitory activities against human nasopharyngeal carcinoma and non-small cell lung tumor cell lines [207]. Synthetic analogues of tasiamide B have been shown to exhibit inhibitory activity against β-secretase 1 which is a potential therapeutic target for Alzheimer’s disease. Docking simulation studies agreed with the structure-activity relationship studies and the results furnished supporting evidence that the analogues can act as β-secretase 1 inhibitors and be developed as drugs for Alzheimer’s disease [208].

Marine microorganisms constitute the greatest sector of marine species and make up over 10% of the total living biomass in the biosphere. They are potential sources of bioactive peptides that may be used as leads in pharmaceutical and nutraceutical developments. However, the biodiversity of marine microorganisms and versatility of their bioactive peptides are usually underestimated and thus far have not been fully explored. From the relatively small number of peptides that have been isolated, only a low percentage has been studied to date for their potential as commercial products. However, as more peptides originating from marine microbes are evaluated at the preclinical and clinical stages, optimization of marine microbe fermentation broth and development of high-throughput culture methodology [209] will provide momentum to and expedite the present investigation on the marine peptides and the findings can be applied in pharmaceutical and nutraceutical industries [210].

## 6. Conclusions

Marine organisms comprise roughly one-half of the total global biodiversity and they constitute a huge and valuable resource of natural bioactive molecules. Marine organisms have gained substantially in importance as a supply of new compounds [211]. Early marine studies were usually conducted in shallow coastal waters since it was a daunting task to sample marine organisms in deep water. General scuba divers can only access habitats at a depth of around 50 m which leaves aside unexplored an enormous population of marine organisms living in the inaccessible environments [212]. With the development of submarines or remotely operated vehicles, these equipments can reach the previously untapped habitat and sample marine organisms in deep waters [213].

Usually a sizeable quantity of raw materials is needed for the purification of marine peptides especially when the peptides of interest are not facile to purify or present in only trace amounts in the marine organisms [189]. It is an obstacle that discourages the investigators from working on marine peptides, and hence a sustainable supply of original marine source is a prerequisite. Fortunately, this hurdle can be circumvented by mariculture (cultivation of marine organisms in the open ocean) or aquaculture (cultivation of marine organisms in artificial conditions). The peptides can also be produced by heterologous peptide expression in cultured microorganisms in combination with fermentation techniques. In addition, chemical peptide synthesis or total synthesis is also feasible for making short marine peptides [13]. All the aforementioned methods can produce the marine sources or the peptides effectively, and with minimal environment impact. Because of their broad spectrum of bioactivities such as antimicrobial, antiviral, antitumor, antioxidative, cardioprotective (antihypertensive, antiatherosclerotic and anticoagulant), immunomodulatory, analgesic, anxiolytic, anti-diabetic, appetite suppressing and neuroprotective activities, marine peptides have potential applications in the pharmaceutical industry and more companies are willing to invest in this area.

Besides, the nutraceutical industry has also progressively been investing more resources in this field and hunting for new possible products. Marine fish protein hydrolysates have drawn much attention due to their diverse biological activities and natural abundance. They came from under-utilized natural resources like the discarded seafood processing wastes (trimmed-off muscle, bones, fins, skin, and gills) and have been regarded as potential materials for the derivation of bioactive peptides for use in nutraceutical industry. Extensive studies of the protein hydrolysates will contribute to the generation of novel bioactive compounds. Thus, they are believed to be a valuable resource for food as well as pharmaceutical and nutraceutical industries. Patients or consumers are more knowledgeable nowadays and conscious about undesirable side effects of drugs and the association between diet and health. This leads to a self-medication mindset such as the desire to avoid chemically synthesized drugs and accept natural food products or nutraceuticals. Although these products show lower efficacy than specific drugs, they do not have the drawback of undesirable side effects. The intake of specific food components or nutraceuticals for prevention and/or treatment of certain diseases are becoming more popular. Marine peptides have high potential nutraceutical and medicinal values because of their effectiveness in both prevention and treatment of various diseases. However, further investigations and clinical trials are needed in order to develop and produce cost-effective and safe drugs from these peptides. Moreover, cost-effective and safe natural health products can be produced from marine bioactive peptides, though further studies and clinical trials are needed.
